# Triterpenes for Well-Balanced Scar Formation in Superficial Wounds

**DOI:** 10.3390/molecules21091129

**Published:** 2016-08-27

**Authors:** Stefan Kindler, Matthias Schuster, Christian Seebauer, Rico Rutkowski, Anna Hauschild, Fred Podmelle, Camilla Metelmann, Bibiana Metelmann, Charlotte Müller-Debus, Hans-Robert Metelmann, Isabella Metelmann

**Affiliations:** 1 Department of Oral and Maxillofacial Surgery/Plastic Surgery, Greifswald University Medicine, Ferdinand-Sauerbruch-Str. DZ 7, Greifswald 17475, Germany; kindlers@uni-greifswald.de (S.K.); schusterm@uni-greifswald.de (M.S.); seebauerc@uni-greifswald.de (C.S.); rutkowskir@uni-greifswald.de (R.R.); anna.hauschild@uni-greifswald.de (A.H.); podmelle@uni-greifswald.de (F.P.); metelmann@uni-greifswald.de (I.M.); 2 Department of Anesthesiology, Anesthesia, Intensive Care-, Emergency- and Pain Medicine, Greifswald University Medicine, Ferdinand-Sauerbruch-Str., Greifswald 17475, Germany; metelmannb@uni-greifswald.de (C.M.); metelmannc@uni-greifswald.de (B.M.); 3 Department of Surgery, Greifswald University Medicine, Ferdinand-Sauerbruch-Str., Greifswald 17475, Germany; charlotte.mueller-debus@uni-greifswald.de

**Keywords:** triterpene, topical betuline gel, acceleration of healing, wound care, scar formation, aesthetic benefit

## Abstract

Triterpenes are demonstrably effective for accelerating re-epithelialisation of wounds and known to improve scar formation for superficial lesions. Among the variety of triterpenes, betuline is of particular medical interest. Topical betuline gel (TBG) received drug approval in 2016 from the European Commission as the first topical therapeutic agent with the proven clinical benefit of accelerating wound healing. Two self-conducted randomized intra-individual comparison clinical studies with a total of 220 patients involved in TBG treatment of skin graft surgical wounds have been screened for data concerning the aesthetic aspect of wound healing. Three months after surgery wound treatment with TBG resulted in about 30% of cases with more discreet scars, and standard of care in about 10%. Patients themselves appreciate the results of TBG after 3 months even more (about 50%) compared to standard of care (about 10%). One year after surgery, the superiority of TBG counts for about 25% in comparison with about 10%, and from the patients’ point of view, for 25% compared to 4% under standard of care. In the majority of wound treatment cases, there is no difference visible between TBG treatment and standard of care after 1 year of scar formation. However, in comparison, TBG still offers a better chance for discreet scars and therefore happens to be superior in good care of wounds.

## 1. Introduction

Good clinical care of wounds aims for well-balanced healing resulting in fast closure of skin lesions and discreet scar formation. Triterpenes are demonstrably effective for accelerating re-epithelialisation of wounds and are known to improve scar formation for superficial lesions [1,2].

### 1.1. Medically Effective Triterpenes from Natural Sources

The outer bark of birch (Betula alba cortex) contains pentacyclic triterpenes, mainly betuline (BE, up to 34%), but also betulinic acid (BA), oleanolic acid (OA), lupeol (LU) and erythrodiol (ER). They can be extracted as a triterpene-rich dry extract (TE) which is able to form a topically applicable oleogel [3,4].

Birch triterpenes have known antiviral, antimicrobial and hepatoprotective pharmacological activities [5,6,7]. BA, OA or BE also have antitumor effects [4,8,9]. These triterpenes show anti-inflammatory activities, as do others such as ER or LU [9,10,11,12], and potential to enhance epidermal permeability barrier recovery [13] and stimulate wound healing [14] via induction of basal cell proliferation [15].

Such properties are of interest in treating skin diseases where a topical application is preferred. Therefore, the bioavailability and toxicity of the birch triterpene extract is of interest. The toxicity of triterpenes is reportedly relatively low. A 600 mg/kg i.p. dose is tolerated well [16,17,18]. The LD_50_ for OA is 1500 mg/kg (i.p.; mouse) [19]. Only a few pharmacokinetic studies have been published. BA was found in various tissues 24 h after i.p. administration (500 mg/kg; mouse) and reached its highest concentration in perirenal fat. Peak serum concentration (4.0 µg/mL) was observed at 0.23 h after application [20]. These findings indicate that triterpenes are of relatively low toxicity, and therefore can generally be used therapeutically.

However, their solubility is low and bioavailability questionable. The solubility in water of OA and BA is only 0.02 µg/mL [21]. In animal experiments triterpenes are administered i.p. or s.c. as a dispersion, whereas i.v. they have to be dissolved. Therefore, organic solvents, for example *N*,*N*-dimethyl-acetamide, are necessary [22], but they are not excipients of first choice for pharmaceutical usage [23]. In contrast, the solubility of BE in oil is approximately 3 mg/mL [4] which offers the possibility of topical application.

### 1.2. Triterpenes for Clinical Use: Topical Betulin Gel

Among the variety of triterpenes, BE is of particular medical interest. As Woelfle and co-workers [24] demonstrated in 2010, highly purified BE promotes keratinocyte differentiation and the terminal differentiation to corneocytes. The clinical relevance of dermally applied BE extract from the outer bark of birch has been evaluated in clinical trials, including 138 patients suffering from actinic keratosis, and investigated in two pilot trials and two clinical trials including 135 patients; the patient population included neurodermitis (33), psoriasis (24), actinic keratosis (28), and laser wound (50) patients [25,26].

Topical betuline gel (TBG) received drug approval in 2016 from the European Commission as the first topical therapeutic agent with the proven clinical benefit of accelerating wound healing (Episalvan^®^, Birken AG, Niefern-Öschelbronn). This ointment of 10% triterpene dry extract from birch cork as the active ingredient and 90% refined sunflower oil only is a semisolid oleogel directly designed for surgical wounds. The active ingredient consists of >80% BE and <20% of the triterpenes, mainly BA, LU, ER, and OA. *N*-Heptane (95%) has been used as extraction solvent.

### 1.3. Accelerating Wound Closure by Triterpenes

The main medical benefit of TBG is acceleration of wound healing, substantiated by an open, blind-evaluated, controlled, prospective, randomized (1:1) phase II clinical trial in patients presenting skin graft wounds [1]. Surgical sites on the upper legs were covered with dressing. TBG was randomly applied to the distal or proximal half of the wound with the other half as untreated intra-individual comparison. The primary efficacy variable was faster re-epithelialization as determined from macro photographs by independent, blinded experts. Twenty-four patients were randomized and completed the trial. After 14 days of treatment, the analysis revealed a highly significant (*p* < 0.0001) superiority of TBG in the primary efficacy variable.

### 1.4. Conditioning of Scar Formation by Triterpenes

As another benefit, TBG treatment of epithelial wounds results in discreet scars, at least demonstrated in ablative laser lesions. A prospective, randomized, controlled experimental trial with blinded analysis intra-individually compared healing of CO_2_-laser wounds treated by TBG to two standard treatment options [27]. The success of healing was evaluated on the basis of aesthetic aspects such as the colour and the texture of the recovering skin related to the surrounding untreated skin.

The experimental laser wounds were made with a CO_2_ laser, resulting in three similar areas of the same size in a line. By randomization, one site was treated with TBG, the second for comparison with a standard moist wound dressing, and the third one by covering with dry gauze compress. TBG was applied to the wound three times daily for 14 days.

The outcome of the three different ways of treatment was evaluated by blinded analysis of photographs, presenting the wound areas in comparison with untreated skin. Remote experts had to score which of the treated areas looked the most like the untreated skin.

A total of 50 human subjects have been involved in this descriptive comparison. 32 of the subjects were male and 18 female, presenting with a median age of 30 years and a skin type mainly Fitzpatrick 2 (52%) and 3 (36%). All subjects reached the end of the 14-day treatment period under observation and 25 subjects were assessed after 10 weeks.

**Figure 1:**
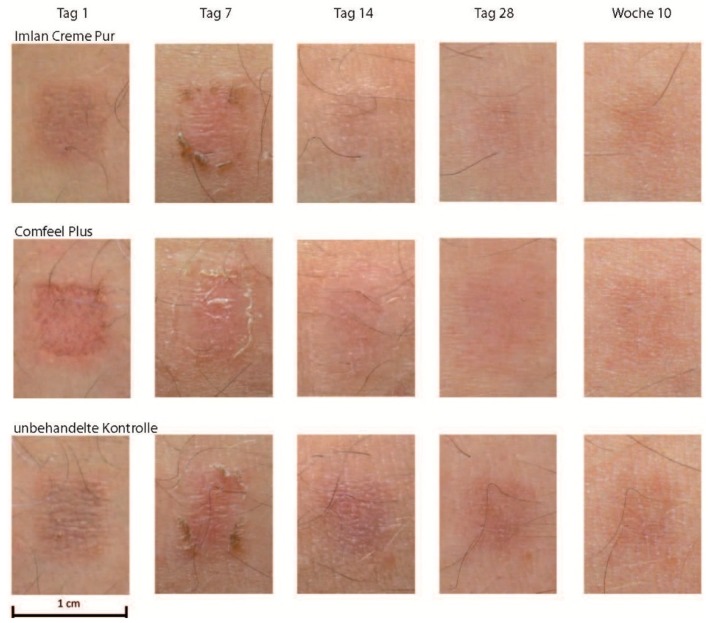
Scar formation of laser skin lesions treated by TBG, hydrocolloid or gauze (one of 50 cases presented). The columns (Tag 1, 7, 14, 28, Woche 10) show the status of healing at days 1, 7, 14, 28 and after 10 weeks, beginning at the left side with day 1 after making 3 laser skin lesions of similar size and depth. The lines (Imlan Creme Pur, Comfeel Plus, unbehandelte Kontrolle) show the 3 different methods of treatment of the 3 similar laser skin lesions, being TBG at the top line, hydrocolloid in the middle, and gauze at the bottom line.

The course of epidermal recovery most commonly observed in this study is illustrated in Figure 1. The first line is demonstrating the lesion treated by TBG and its aspect developing similarity to normal skin around 14 days. In direct comparison, this area is recovering faster than the area under the influence of hydrocolloid dressing, presented in the second line with a slightly poorer result in terms of redness and texture after 14 days. The treatment of the laser lesion by just covering with gauze, as to be seen in the third line, never produces results as convincing as the other treatment options.

### 1.5. Does Accelerated Healing Accompany Discreet Scars?

Clinical investigations have been performed comparing different types of wound dressings as well as metabolic supplementation [28], growth hormones [29], physical measures like extracorporeal shock waves [30,31], cold physical plasma at atmospheric pressure [32] and traditionally used natural topical wound healing substances like comfrey, honey, or aloe vera [33,34,35]. However, to date, no generally accepted treatment other than TBG exists for surgical superficial wounds that demonstrates proven acceleration of superficial wound healing.

Rapid epithelization of superficial wounds reduces discomfort for patients, pain and risk of infections caused by unfortunate outcome chronification of wounds and unaesthetic scars [36]*.* In cosmetic surgery, accelerated aesthetic recovery reduces the post-operative down time of the patients, but more important is the long term benefit of discreet scars. As discreet scar formation matters in any case, the question remaining is whether acceleration of healing is actually followed by aesthetic scars.

## 2. Results and Discussion

Two self-conducted recent clinical studies with a total of 220 patients involved in TBG treatment of surgical wounds [1,37] have been screened for data concerning the aesthetic aspect of wound healing. Study protocols were approved by the ethics committee, and the studies were performed in compliance with the International Conference on Harmonization guidelines for Good Clinical Practice and the principles in the Declaration of Helsinki (EudraCT nos. 2012-003390-26 and 2012-000777-23).

### 2.1. Investigating Scar Formation by TBG in Skin Grafting Wounds

Based upon open, blindly evaluated, prospective, controlled, randomized, multi-centre clinical trials, the aim of data screening was to assess scar formation after 3 months and 12 months post-operatively. Primary objective was the intraindividual aesthetic difference of two wound halves caused by skin grafting at the upper leg, treated with either a standard of care moist wound dressing alone or TBG. The assessment was based on photo evaluation by a remote panel of blinded experts and personal evaluation of the patient.

Prior to skin grafting, the donor site was divided into two equal halves randomized 1:1 for either (i) TBG combined with a non-adhesive moist wound dressing or (ii) a wound dressing alone. TBG was applied approximately 1 mm thick to the appropriate wound half on the wound-facing side of the dressing or directly onto the wound. Every 3 or 4 days according to protocol or more frequently if medically necessary, the wound dressing was changed, the wound was cleaned, and medication was applied for the appropriate wound half. Treatment continued up to complete closure of both halves of the wound or, if complete closure of both wound halves was not observed, until day 28.

For observer-blinded aesthetic analysis of wound closure, photos were taken for evaluation of long-term outcome 3 and 12 months after treatment. Only photos confirmed to be free of markings were considered for a read of healing result. Eligible photographs were independently evaluated by wound experts (experienced surgeons or dermatologists) via a web-based electronic blinded read tool. Long-term outcomes were estimated for determination of which wound half was most similar to surrounding healthy tissue in terms of texture, hair growth, pigmentation, and redness.

For the unblinded analysis, patients estimated directly their aesthetic appreciation after 3 and 12 months by use of questionnaires about the efficacy and tolerability of the two treatments on a 5-point Likert scale (which treatment was more efficacious/which treatment was better tolerated/which treatment resulted in better aesthetics of scar formation) [38,39].

### 2.2. Typical Clinical Course of Scar Formation

**Figure 2:**
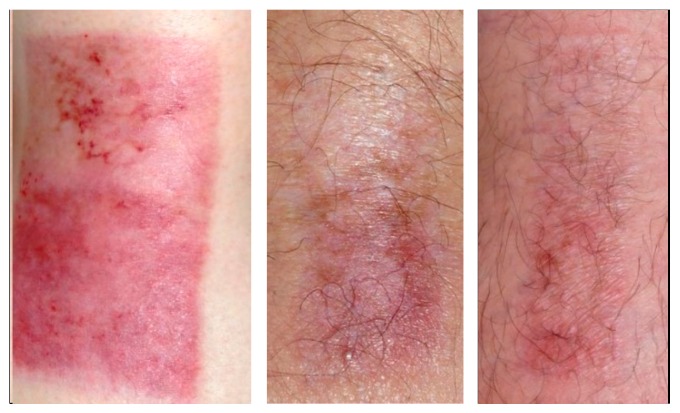
Skin graft wound, separated in two halves, treated with TBG at the upper half and standard of care at the lower half, left photo showing situation of healing 14 days postoperatively, middle photo showing situation of healing 3 months postoperatively, and right photo showing situation of healing 12 months postoperatively [27].

The course of wound healing most commonly observed in the study is illustrated in Figure 2. The upper half of the wound has been treated with TBG covered by a dressing, and the lower half has been just covered by the same kind of dressing as standard of care, but here without TBG underlying. The whole wound is known for high absorption of actives, but one half of the wound, decided by randomization, is not in contact with TBG, in this case the lower half as the reference site, and the other half of the same anatomical site is richly supplied with TBG, this time the upper half as the treated site.

After 14 days of treatment (Figure 2, left side) a thin layer of newly developed epithelium covered the site of TBG treatment. After 3 months of healing (Figure 2, in the middle) the epithelium at the TGB site appeared more stable than the other site. After 1 year of healing (Figure 2, right side) the treatment result at the TBG site looked ideal from an aesthetic point of view, while the other site still presented more of erythema.

### 2.3. Variety of Scar Formation

**Figure 3:**
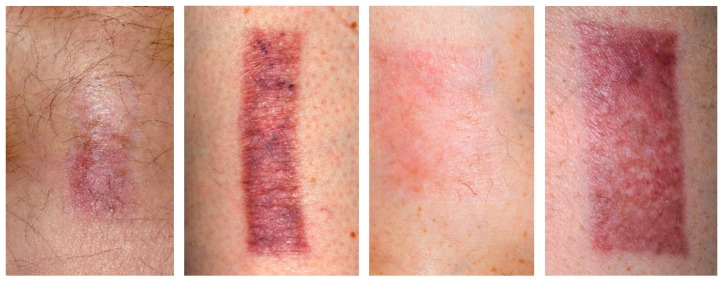
Four different skin graft wounds from four different patients, showing an intra-individual comparison of TBG-treatment (upper half) and standard of care (lower half) for four different results of scar formation [27].

Some cases of TBG treatment (Figure 3, upper half of the former wounds) demonstrated very good long term benefit (Figure 3, left side) in comparison with the site treated by standard of care (Figure 3, lower halves of the former wounds). Other cases demonstrated the superiority of TBG treatment (Figure 3, neighboring the left side), though the aesthetic benefit was poor. In a few cases the effect of TBG and standard of care seems to be almost equal (Figure 3, neighboring the right side)) and in very rare cases TBG treatment resulted in a slightly inferior aesthetic result for the patient (Figure 3, right side).

### 2.4. Scar Formation in Progress (3 Months)

After 3 months of scar formation there were 220 patients evaluable for aesthetic assessment with 220 wound sites, treated in one half with TBG and in the other half with standard of care. 79 of 220 patients enjoyed more discreetly pigmented scars due to TBG. 23 of 220 patients presented better aesthetic results in terms of pigmentation related to standard of care. The remaining patients did not demonstrate any obvious aesthetic differences comparing the TBG and standard of care treated former wound halves. Referring to redness, texture and hair growth TBG was of equal quality to standard of care, though on a somewhat lower level.

A total of 88 patients were asked for their personal assessment of aesthetic benefit. From the patients′ point of view, 42 of 88 decided for better-looking scars, discreet and more similar to normal surrounding skin, when treated with TBG, compared to 9 of 88 patients under standard treatment of care. As aesthetic benefit is subjective and difficult to calculate numerically, this descriptive comparison is not appropriate for statistical evaluation and confirmation by *p*-values.

### 2.5. Matured Scars (12 Months)

**Figure 4:**
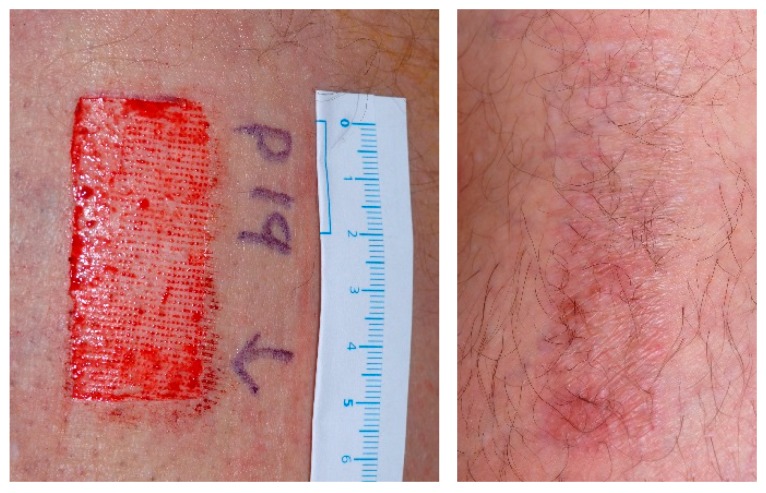
Typical clinical aspect of fresh skin graft wound just before starting TBG-treatment (upper half of the surgical site (left side) and 12 months postoperatively as clinically mature scar (right side) [27].

After 1 year of scar formation there were 158 patients still evaluable for aesthetic assessment with 158 wound sites, treated in one half with TBG and in the other half with standard of care. 39 of 158 patients enjoyed more discreetly pigmented scars due to TBG (Figure 4). 18 of 158 patients presented better aesthetic results related to standard of care. However 101 of 158 patients did not demonstrate any obvious aesthetical differences in aesthetic scar formation comparing TBG and standard of care. Same relations were apparent on lower level of numbers for TBG and standard of care referring to redness, texture and hair growth.

A total of 46 patients were asked for their personal assessment of aesthetic benefit after 1 year. From the patients´ point of view 12 of 46 decided for better looking scars, discreet and more similar to normal surrounding skin, when treated with TBG, compared to 2 of 46 patients under standard treatment of care. In the majority of cases, obviously there is no aesthetic difference visible after 1 year of scar formation between TBG treatment and standard of care. However in comparison with standard of care, TBG offers better chances for discreet scars and happens to be superior in good care of wounds. As aesthetic benefit is subjective and difficult to calculate numerically, again this descriptive difference is not appropriate for statistical evaluation and confirmation by *p*-values.

### 2.6. Discussing Triterpenes as the Active Ingredient of TBG

TBG plus dressing was compared to standard of care alone and not to the vehicle (i.e., pure sunflower oil) as a control because the active ingredient, i.e., birch bark extract TE, changes the viscosity of sunflower oil to such an extent that pure oil would not be comparable. TE therefore functions as an active ingredient and as a galenic excipient at the same time, with a concentration of 10% yielding the optimal viscosity for application on wounds. At a 10% concentration, the consistency is comparable to that of petrolatum. Therefore, a vehicle control is unable to blind the treatment regime. Furthermore, pure, liquid sunflower oil on an adjacent wound area would spread over to the test area and the TE would diffuse the other way, jeopardizing the test design. It is unlikely that sunflower oil alone would improve the conditions of wound healing. In an ex vivo porcine ear punch biopsy model, sunflower oil or sunflower oil thickened with ethyl cellulose rather slowed down wound healing, while the addition of TE to either medium accelerated the wound healing significantly. In rats orally administered sunflower or other nutritional oils, wound closure was delayed [40]; ozonized sunflower oil improved wound healing merely because of its antimicrobial activity [41]. The pharmacological properties of birch bark triterpenes, however, may well explain the acceleration of wound healing. Woelfle et al. [24] found that TE enhances the expression of differentiation markers like involucrin and keratin-10 in early confluent human primary keratinocytes. An upregulation and indirect activation of TRPC6 via high [Ca ++] ex by TE was postulated [42].

### 2.7. Discussing TBG Subject to Wound Dressing Procedure

TBG may be used in combination with covering dressings (closed approach) or without (open approach). In the laser lesions presented, TBG has been administered with an open approach, while in the skin graft wounds TBG was covered.

The open approaches consist of the application of external substances without an occlusive cover to promote wound healing, to reduce side effects and to optimize the functional and aesthetic result. They are easy to apply and not disfiguring for the patient. Most authors recommend bland ointments or creams to help avoid crusting from serous exudate and to minimize skin irritations or allergic reactions [43]. Topical steroids should not be used due to an increased risk of wound infections [44]. The importance of a peri- or postoperative antibacterial prophylaxis is controversial. Antibiotic prophylaxis is useful but not essential, because meticulous wound care and close clinical monitoring of patients daily with routine bacterial swabs can detect infection early [45]. Other studies even show that a significantly higher rate of infection occurred in patients receiving combination intraoperative and/or postoperative antibiotic prophylaxis [46]. The advantage of open wound management is that the wound is visible and there is no cover under which bacteria can grow undetected.

Closed approaches, on the other hand, use semi-occlusive or occlusive dressings to cover the wound area. Examples are a semi-occlusive dressing of polyurethane film and an occlusive hydrocolloid dressing (HCD). The wounds treated with HCD or polyurethane film healed significantly faster than those covered with fine mesh gauze or silver sulfadiazine [47]. There are studies which report a better re-epithelialization by preventing wound desiccation with closed approaches [48]. Skin biopsies have been examined at multiple time points following resurfacing, and re-epithelialization begins after 48 h in skin that has been occluded. Skin that has been left open with no treatment forms has no keratinocyte migration after 48 h, thus displaying delayed wound healing [49]. Lower infection rates are generally achieved despite bacterial proliferation under some occlusive dressings [50]. Proper postoperative dressing changes may reduce the incidence of infection. One big disadvantage of a closed regimen is the aesthetically disturbing dressing which can be depressing for the patients, especially when used in the face. In terms of aesthetic benefit Innes and co-workers [51] have demonstrated peculiar differences f. e. in moist wound dressing material. Markl and co-workers [52] however did not observe significant differences regarding quality of healing and scar formation.

In any case, direct comparisons of healing times from different studies are critical with respect to different kinds of wounds dressings and control treatments. While remarkable differences between different donor site dressings are reported in specific comparative studies [53] a comprehensive systematic review of 72 trials could not find clear results the superiority of either moist or dry dressings in any parameter except pain, since there are so many factors influencing outcomes [54].

To sum up, the open approaches are easy to administer and are not very disturbing for the patient whereas the closed approaches can lead to a better wound healing but for the price of visual disadvantages. This led to our study design: we chose the closed approach, which, regarding the literature, should lead to a better wound healing compared to open approaches and has to be seen today as standard of care.

## 3. Material and Methods

### 3.1. Materials

TBG (Episalvan^®^, Birken AG, Niefern-Öschelbronn, Germany) is a semisolid oleogel of 10% triterpene dry extract from birch cork as the active ingredient and 90% refined sunflower oil only. The active ingredient consists of >80% betuline and <20% of the triterpenes, mainly betulinic acid, lupeol, erythrodiol, and oleanolic acid. *N*-Heptane (95%) has been used as extraction solvent.

A siliconized foam dressing (Mepilex^®^, Mölnlycke Health Care, Goteborg, Sweden) was in use as standard-of-care dressing.

### 3.2. Methods

The intraindividual clinical comparison of two wound halves caused by skin grafting at the upper leg, treated by just a standard of care dressing or by the same dressing combined with TBG, and the assessment of outcome based on photo evaluation by a remote panel of blinded experts and personal evaluation of the patient is a well-established method [1,2].

Briefly, the wound site was divided into two equal halves randomized 1:1 for either (i) TBG combined with a non-adhesive moist wound dressing or (ii) a wound dressing alone. TBG was applied approximately 1 mm thick to the appropriate wound half on the wound-facing side of the dressing. Every 3 or 4 days according to protocol or more frequently if medically necessary, the wound dressing was changed, the wound was cleaned, and medication was applied for the appropriate wound half. Treatment continued up to complete closure of both halves of the wound or, if complete closure of both wound halves was not observed, until day 28.

For observer-blinded aesthetic analysis of wound closure, photos were taken for evaluation of long-term outcome 3 and 12 months after treatment. Eligible photographs were independently evaluated via a web-based electronic blinded read tool. Long-term outcomes were estimated for determination of which wound half was most similar to surrounding healthy tissue in terms of texture, hair growth, pigmentation, and redness.

For unblinded analysis, patients estimated directly their aesthetic appreciation after 3 and 12 months by use of questionnaires.

## 4. Conclusions

Good clinical care of wounds is aiming at well-balanced healing. Fast closure of skin lesions and discreet scar formation is the visible outcome of well-balanced healing. In plastic surgery and when evaluating the benefit of good scar formation, it is difficult to provide quantitative data for comparative analysis. Reporting the wound area healed or scarred does not hit the point, since the patients’ subjective view is the only parameter of success. Their appreciation many times is the most important and in aesthetic operations even the only aspect that matters in terms of a discreet scar formation.

TBG as a medical application of triterpenes is known for accelerating re-epithelialisation of wounds [1,2]. Another benefit of TBG is to improve the appearance of scar formation in superficial lesions*.* Three months after surgery wound treatment with TBG results in more discreet scars in about 30% of cases, while standard of care produces discreet scars in about 10%. Patients themselves appreciate the results of TBG after 3 months even more (about 50%) compared to standard of care (about 10%).

One year after surgery, the superiority of TBG counts for about 25% in comparison with about 10% under standard of care, and from the patients′ point of view for 25% compared to 4% under standard of care. In the majority of wound treatment cases, there is no difference visible between TBG treatment and standard of care after 1 year of scar formation. However in comparison with standard of care, TBG still offers better chances for discreet scars and is therefore superior for good care of wounds.
